# Transfer of ^137^Cs into Maize (*Zea mays* L.) at Different Growth Stages in the Area Contaminated by the Chernobyl Fallout

**DOI:** 10.3390/toxics14080714

**Published:** 2026-08-12

**Authors:** Tatiana Paramonova, Olga Denisova, Natalia Kuzmenkova, Leonid Turykhin, Maria Godyaeva, Alexei Konoplev

**Affiliations:** 1 Laboratory of Geochemistry of Natural Waters, Geography Faculty, Lomonosov Moscow State University, Moscow 119991, Russia; denisovaoe@my.msu.ru (O.D.); kuzmenkovanv@my.msu.ru (N.K.); turykhinla@my.msu.ru (L.T.); airrune@yandex.ru (M.G.); alexeikonoplev@gmail.com (A.K.); 2 Faculty of Soil Science, Lomonosov Moscow State University, Moscow 119991, Russia; 3 Chemistry Faculty, Lomonosov Moscow State University, Moscow 119991, Russia; 4 Institute of Environmental Radioactivity, Fukushima University, Fukushima 960-1296, Japan

**Keywords:** radiocaesium, Chernobyl, root uptake, bioaccumulation, transfer factor, seasonal variations, maize

## Abstract

Accumulation of radionuclides in crop products can pose public health risks. Parameters of ^137^Cs root uptake by maize were investigated at four growth stages, from leaf development to ripening (June–September), in the post-Chernobyl area of the Plavsk radioactive hotspot (the Tula region of Russia). It was established that the chernozem of the agrosystem still contains about 180 kBq ^137^Cs m^−2^. Seasonal trends of ^137^Cs activity concentrations in above- and belowground parts of maize differ appreciably. In aerial parts, the ^137^Cs accumulation rate increases as vegetative organs develop from June to July, then drops when generative organs appear in August. In the belowground biomass, the ^137^Cs content increases with the growth of fine roots, which serve as relative concentrators of the radionuclide. However, at all stages of growth, radionuclide transfer to maize occurs with low intensity. It does not directly depend on changes in biomass, dry matter content, or ^40^K content, and only slightly relates to the ash content. The transfer factor is estimated to be 6.2 × 10^−3^ for cob kernels (grain) and 3.8 × 10^−2^ for stems and leaves, which corresponds to the IAEA-recommended values and ensures acceptable levels of ^137^Cs accumulation in crop products produced in the territory.

## 1. Introduction

One of the most serious pathways of agricultural land contamination is atmospheric fallout following accidents at radiation-related facilities. Typically, contamination covers large areas and is long-term, which creates difficulties in the use and rehabilitation of affected croplands. In particular, after the Chernobyl Nuclear Power Plant accident in 1986, radioactive contamination of soil with the main dose-contributing radionuclide ^137^Cs in excess of 37 kBq m^2^ was recorded in Europe over an area of about 215,900 km^2^, of which 60,000 km^2^ was in the European part of Russia alone, including >23,000 km^2^ of agricultural land [1]. A revision of the radioecological state of these arable lands after 30 years (which corresponds to a half-life of ^137^Cs with T_1/2_ = 30.17 years) showed that the area of cropland remaining radioactively contaminated had decreased by only 32–47% [2], depending on the landscape and cropping features in different regions, with the major part of the radionuclide still concentrated within the arable horizon [3,4]. Nowadays, 40 years after the Chernobyl accident, the spread of ^137^Cs along the food chain “agricultural soils–crops–(farm animals)–humans” still poses certain risks to public health, but at the same time, well-detectable quantities of the radionuclide make it possible to clarify migration patterns in the natural conditions of post-Chernobyl territories.

Cereals are usually the reference for other agricultural crops, as they form the basis of a field rotation under different bioclimatic conditions and zones. Moreover, maize, wheat, and rice are considered by FAO to be among the world’s essential human food staples and key elements of global food security, accounting for ≈40% of calories and protein in food consumption [5,6]. Maize (*Zea mays* L.) is a leading cereal crop widely cultivated in various regions and countries over an area of ≈200 million hectares, with the highest productivity (≈6000 kg ha^−1^) among cereals and annual global production of ≈ 1.1 billion tons [6,7]. Belonging to the Gramineae family, maize differs from other cereals in C4 photosynthesis, high rates of metabolism and biomass growth, and the specific architecture of roots and shoots, which distinguishes it as a separate agro-industrial group. It is a multipurpose crop, used mainly as feed and food, and also partly for non-food purposes [6]. In the human diet, fresh, boiled, frozen, or canned kernels, maize flour and meal, as well as processed maize products (corn flakes, etc.), are valued for their high starch content, essential mineral elements, and B vitamins [8].

However, the nutritional value of maize for humans and livestock is only realized if food and feed are environmentally safe for consumption. Maize is considered to be sensitive to abiotic stresses, including metal toxicity [9]. In turn, contamination of food products with heavy metals and radionuclides can negatively impact human health and is a serious agricultural problem in many countries [10]. Generalized IAEA analysis of ^137^Cs transfer to maize grain, stems, and shoots in radioactively contaminated territories, conducted for temperate, subtropical, and tropical climate zones, shows that the intensity of biological migration of the radionuclide in the “soil–maize” system is somewhat higher than for cereals in general, and, in addition, increases significantly under hot, arid climatic conditions [11,12]. It appears that quantitative assessment of the intensity of ^137^Cs accumulation in maize based on the predictor coefficient (transfer factor, TF) is extremely important from both theoretical and applied perspectives. Although the TF is not a complete constant even for a fixed “soil–plant” pair, it is currently the main tool for estimating the intensity of ^137^Cs root uptake according to the phylogenetic model of radionuclide bioavailability [13,14,15].

The question arises: to what extent does the accuracy of TF estimation depend on the stage of crop phenological development at which the accumulation of ^137^Cs in biomass is accounted for? There is not much information that sheds light on this problem, especially in the context of field research on radioactively contaminated land. However, there are reasons to consider seasonal variations in the intensity of root uptake of ^137^Cs as a significant factor in TF variability [16,17,18]. Seasonal changes in ^137^Cs activity concentrations in leaves were observed for macrophytes of Glyboke Lake in the Chernobyl Exclusion Zone [18], as well as in coniferous and deciduous broadleaf trees in the radioactive contamination zone around the Fukushima Daiichi Nuclear Power Plant [19]. A decrease in TF was shown as the fruiting bodies of the *Boletus edulis* grew [20]. In field model experiments, significant variability in TF was shown depending on the growth stage of the agricultural crop, and in the case of cereals (wheat, brown rice), the trends in ^137^Cs accumulation in straw and grain had different temporal fluctuations [21]. In contrast, in another pot experiment, a synchronous increase in TF values was found in both brown rice grain and its straw as plants grew, and the trend was maintained for root uptake of ^137^Cs from soils with different properties [22]. At the same time, some field studies did not reveal any regular changes in ^137^Cs activity concentration in plants of the Ericaceae and Juncaceae families, but revealed seasonal trends for a member of the Cyperaceae family depending on the ontogenesis stage [23]. Thus, there is no common understanding of the patterns of changes in ^137^Cs activity concentration in crop organs at different stages of their growth or of the significance of seasonal changes in TF for predicting the intensity of transport of radionuclides from soil to plant.

The aim of the present study was to determine the intensity of root uptake and to quantify ^137^Cs fluxes into maize at different growth stages of plants. The study was conducted to elucidate some controversial and applied aspects of biogenic migration of ^137^Cs: (i) the possibility of intra-seasonal dynamics of ^137^Cs transfer to maize at different stages of plant ontogenesis; (ii) ^137^Cs *intra-planta* bioaccumulation patterns with consideration of the radionuclide distribution over plant organs as they differentiate and develop; (iii) the specificity of ^137^Cs root uptake by maize compared to the total flux of mineral nutrients and potassium (by ^40^K) into the plant; (iv) the value of TF in the “arable clayey soil–maize” system under semi-arid ambient in the remote period after accidental radionuclide fallout; (v) the food hygienic characteristics of maize products from the long-term radioactively contaminated area.

It is assumed that solving these questions can supplement the general understanding of the processes of root uptake and biological migration of ^137^Cs in agrosystems located in the areas of major radioactive fallout.

## 2. Materials and Methods

### 2.1. Study Area, Soil and Crop Characteristics

Field observations of seasonal variability in ^137^Cs root uptake characteristics by maize were conducted in 2021 in the area of pronounced Chernobyl fallout formed in the southern part of the Tula region of Russia (≈650 km northeast of the Chernobyl Nuclear Power Plant)—the so-called “Plavsk radioactive hotspot” (PRH). The region historically specialized in agriculture; currently, 85% of its territory is occupied by agricultural land, of which more than 95% is cropland [24]. In 1986, the PRH lands were contaminated with ^137^Cs at a level of 185–555 kBq m^−2^, which was 5–15 times higher than the state standard [1]. At present, the farmlands are still significantly polluted with ^137^Cs, exceeding the permissible level of its accumulation in the soil by 4–6 times [4,25]. An old plowed horizon (Aop) is ubiquitous in the territory, indicating deep tillage to a depth of 30 cm after the Chernobyl fallout as a countermeasure to reduce the root uptake of ^137^Cs by crops [4]. Soils of the PRH area are mainly represented by arable Luvic Chernozems with high humus content (6.8 ± 0.2% of C_org_), neutral pH _H2O_ (6.6 ± 0.1), bulk density of 1.1–1.2 g cm^−3^, K_tot_ 2.0 ± 0.1%, K_exch_ 235 ± 54 mg kg^−1^, a clay loam texture (clay 42.0%, silt 57.8%, sand 0.2%), and mineral composition enriched in clay minerals of the illite and montmorillonite groups [4,26]. Soils with such properties have a pronounced ability to selectively sorb ^137^Cs in the interlayer space of clay minerals [27,28], thereby reducing its bioavailability, as only a small amount of the radionuclide is mobile and capable of passing into the soil solution and being taken up by plants. However, the downside of the strong binding of radiocaesium to the soil matrix is its accumulation in the surface root zone for very long periods.

**Figure 1:**
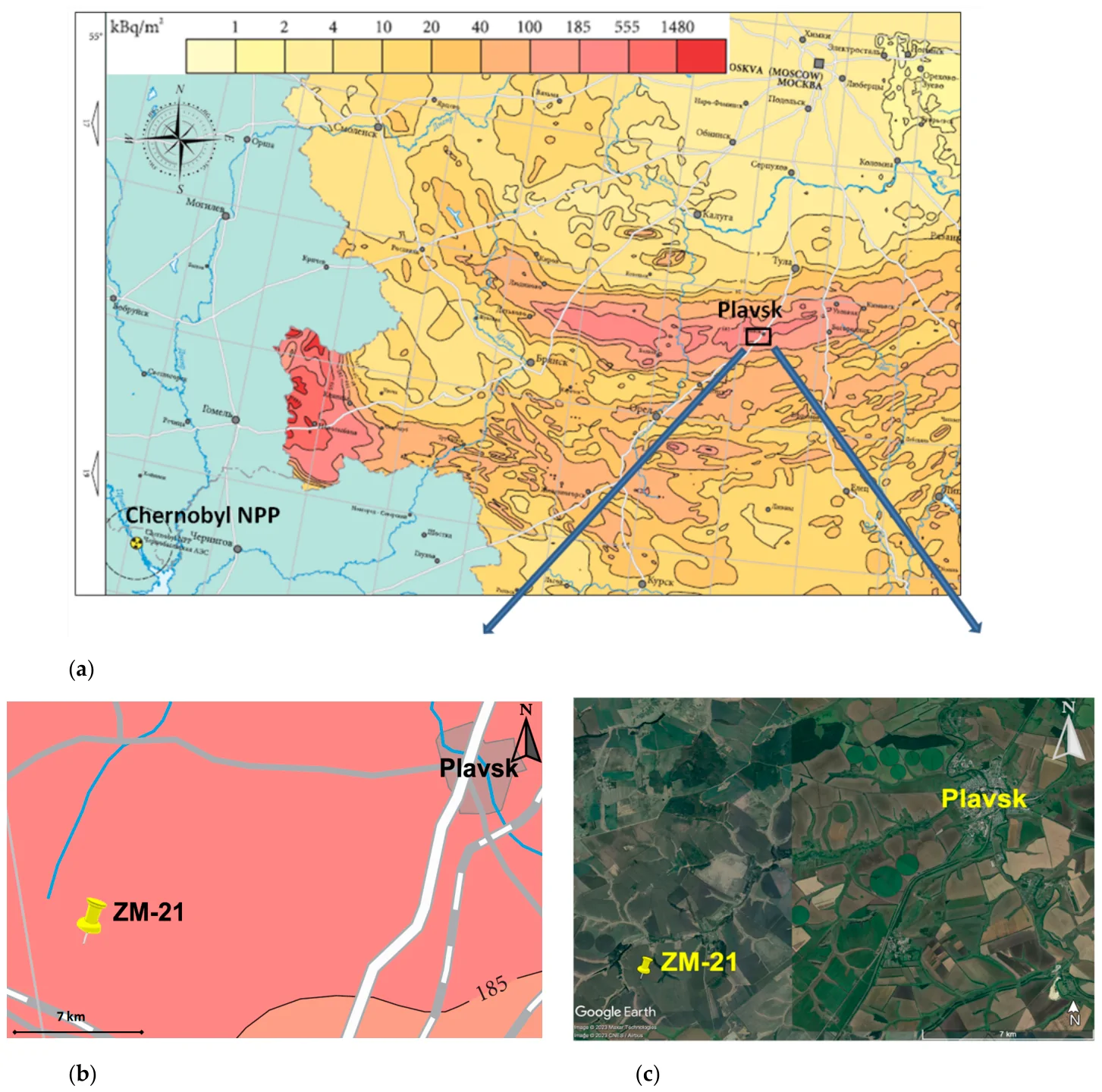
Location of the PRH area and experimental plot with maize agrosystem (point ZM-21): (**a**) and (**b**) on the map of generalized levels of the ^137^Cs pollution in Europe after the Chernobyl accident, May 1986, with the scale of soil contamination with ^137^Cs [1]; (**c**) on the Google Earth image.

The experimental plot was situated in the central part of the PRH area (Figure 1). It was located on the non-eroded middle part of the interfluve slope, with a gradient of 2^o^ and an absolute altitude of 237 m above sea level. In 2021, the place in the crop rotation on this plot was occupied by Pioneer maize grain hybrid P7515 (170 by FAO classification—early maturing), which is characterized by drought tolerance, disease tolerance, and high yield potential. In the autumn preceding maize sowing, the chernozem soil was plowed to a depth of ≈20 cm (Ap″ layer) and 60 kg ha^−1^ of P and K fertilizers (in terms of the active ingredient) were applied. In spring, when sowing at the end of the second decade of May, the soil was cultivated to a depth of 6–8 cm (Ap′ layer) and 144 kg ha^−1^ of N fertilizer was applied. The seeding rate was 70–80 thousand units ha^−1^, with a row spacing of 70–75 cm; seeds were placed 15–20 cm apart in the row.

### 2.2. Sampling and Processing

Simultaneous soil and crop sampling in the maize agrosystem was carried out monthly from 19 June to 18 September 2021, at approximately equal intervals. Weather conditions in the study area in 2021 did not differ much from the long-term averages, with an average summer air temperature of +16.8 °C (+1–+34.1 °C) and precipitation of 311 mm (63 days with precipitation, mostly in May and September, and 60 days without precipitation, mostly in July and August) during the growing season, according to the Plavsk weather station.

The sampling dates corresponded to the following stages of phenological development of maize on the international BBCH scale: June—leaf development (codes 14–15, 4–5 true leaves unfolded); July—stem elongation (codes 32–34, 3–4 nodes detectable); August—development of fruit (codes 71–73, beginning of kernels development and early milk); September—ripening (codes 87–89, physiological maturity and fully ripe) [29]. The listed stages of maize growth are hereafter referred to as S1–S4, respectively.

**Figure 2:**
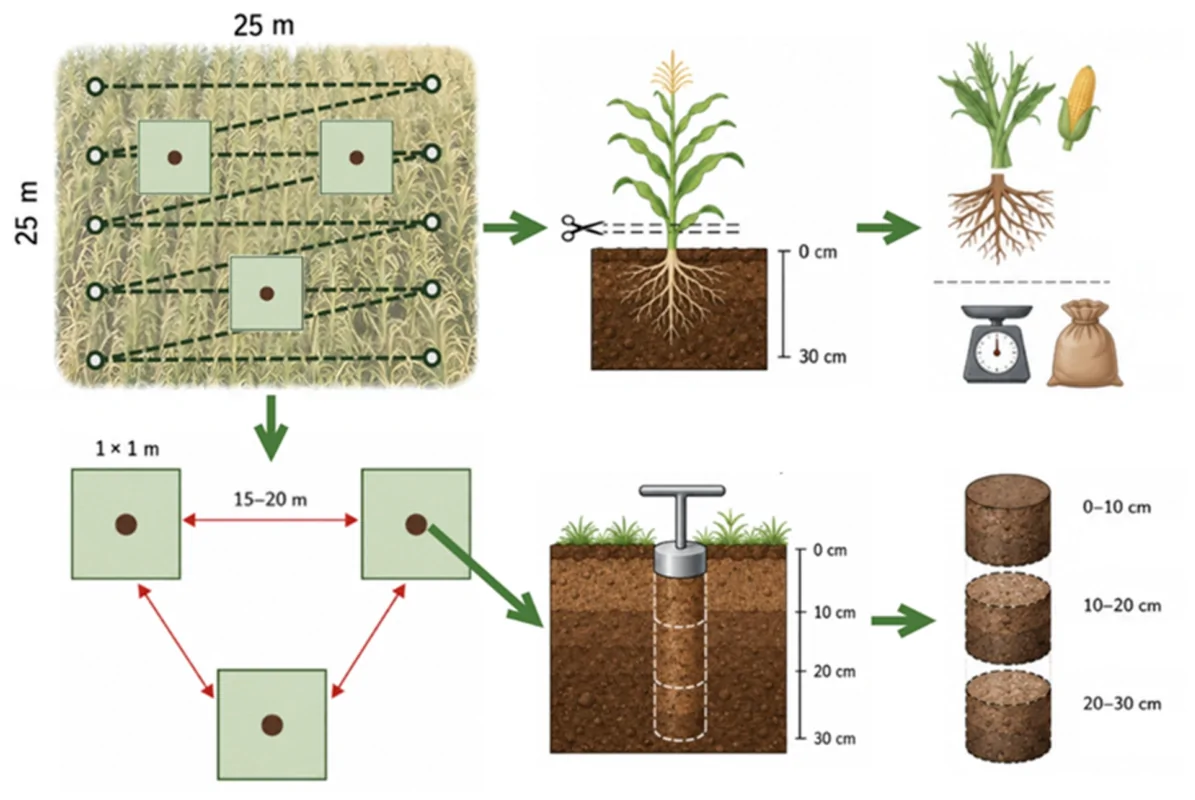
Schematic representation of the soil and plant sampling procedure.

At each observation period, 3 points were selected on a homogeneous reference site of ≈625 m^2^ within the experimental plot, where soil and vegetation characteristics were recorded in combination with each other (Figure 2). The aerial part of the biomass was taken from a fixed area of 1 × 1 m and manually clipped to a height of 2 cm from the ground surface to minimize external contamination of the shoots. As a rule, a sample of the aboveground part of the biomass, taken from the recording area, was made up of 14–16 maize plants. The roots remaining in the soil were taken from the same area to a depth of the main rooting zone (Ap′ + Ap″ + Aop horizons, ≈30 cm). Plant height and root length were determined at the time of sampling. In addition, large samples of both above- and belowground parts of plants (10–14 kg) were collected over the experimental plot along Z-shaped routes in order to separate various organs and fractions of maize biomass in sufficient quantities and ensure the accuracy of subsequent γ-spectrometric analysis. At the S1 growth stage, plants were divided into above- and belowground parts only; as maize developed and its vegetative and generative organs formed, the biomass fractionation was increasingly detailed. At growth stages S3 and S4, the following individual biomass fractions were collected: stems, leaves, cob husks, cob kernels, cob stalk, tassels; root necks, brace roots (5–10 mm), medium-sized seminal and lateral roots (1–3 mm), and fine roots (<1 mm).

Soil sampling was carried out in a stratified random manner in 3 replicates [30]. Soil core samples were taken from a depth of 30 cm in 10 cm increments using an 8 cm diameter steel cylindrical sampler. Each time, the depths of the soil horizons within the strata sampled were described. In fact, the upper 30 cm soil layer taken into study represented a system of cultivated, plowed, and old-plowed horizons (Ap′ + Ap″ + Aop), within which the main inventories of ^137^Cs were concentrated.

After transfer to the laboratory, aboveground biomass fractions were weighed for fresh biomass determination and washed under running water. The maize roots were repeatedly and thoroughly washed free of soil particles under a head of water on a system of screens with mesh sizes of 1 mm and 0.25 mm until the washing water was completely clear. The thinnest roots that passed through the 0.25 mm sieve were collected quantitatively in a pan, washed by multiple decantation, and then combined with the biomass of fine roots. The fractions of maize roots were then air-dried and weighed to determine the fresh biomass. Finally, plant samples were dried at 75 °C for 24–48 h, weighed for dry biomass determination, and then ground in a special mill.

Soil samples were dried at room temperature, weighed, ground using an agate ball mill, and sieved to particles ≤1 mm. Soil moisture and bulk density were recorded during sample processing.

### 2.3. Laboratory Analyses

Determination of the γ-emitting radionuclides—technogenic ^137^Cs and naturally occurring ^40^K—in soil and crops was carried out using a Canberra γ-spectrometer (Mirion Technologies, Atlanta, GA, USA) with an ultrapure germanium (HPGe) semiconductor detector. Analysis of ^137^Cs was of direct and primary interest for this study; the content of ^40^K in arable chernozem and maize organs was determined for comparison of seasonal trends in root uptake of both radionuclides. The mean estimates of the activity concentration of radionuclides and other monitored parameters were obtained on the basis of three independent samples corresponding to three sampling repetitions.

In addition, for the combined sample of the main soil rooting zone (Ap′ + Ap″ + Aop horizons), the proportions of water-soluble, exchangeable, mobile, acid soluble, and residual forms of ^137^Cs fractions were determined using the sequential extraction method [31].

The detection efficiency of ^137^Cs (661.5 keV) was 2.5%; the detection efficiency of ^40^K (1460.8 keV) was 1.2%. The analytical error of γ-spectrometry did not exceed 2%.

Supplementary soil and maize properties (soil moisture and bulk density, dry matter and ash content in biomass fractions) were determined in 3–4 replicates by conventional techniques. All the main characteristics and concentrations of ^137^Cs activity in plants were expressed on a dry mass basis.

### 2.4. Calculations and Statistics

To estimate the seasonal variations in the intensity of ^137^Cs root uptake from radioactively contaminated arable chernozem by maize, the values of TF were calculated as follows (1):

TF_tot_ = A_plant_/A_soil_, (1)

where A_plant_ was the activity concentration of ^137^Cs in total maize biomass or in its fraction (Bq kg^−1^), and A_soil_ was the weighted average activity concentration of ^137^Cs in the rooting zone 30 cm deep (Bq kg^−1^).

The process of ^137^Cs transfer from roots to shoots in maize was evaluated by the translocation coefficient (TLC) calculated as (2):

TLC = A_aerial parts_/A_roots_, (2)

where A_aerial parts_ and A_roots_ were activity concentrations of ^137^Cs in the respective above- or belowground biomass fractions (Bq kg^−1^).

Potential phytoremediation ability (PPA) was defined as follows (3):

PPA = (A_plant_ × M_plant_)/(A_soil_ × M_soil_) × 100 (%), (3)

where M_plant_ and M_soil_ were the dry mass of maize and the rooting zone of soil, respectively, normalized per 1 m^2^.

The obtained data were analyzed using the Microsoft Excel 2003 (Microsoft Co., Redmond, WA, USA) and Statistica 8.0 (Statsoft, Tulsa, OK, USA) software packages using basic descriptive statistics and one-way analysis of variance (ANOVA) with Tukey’s posterior test. Factor analysis was carried out based on the principal component analysis (PCA) method with a selection of 2 significant load factors according to the scree test with an explanation of 88% of the variance. Statistical significance was assumed at α < 0.05.

## 3. Results and Discussion

### 3.1. Current Characteristics of ^137^Cs Accumulation in Arable Chernozem

The assessment of the current ^137^Cs accumulation in the soil of the maize agrosystem in the PRH area revealed that, on average, it is 4.6 times higher than the permissible level of 37 kBq/m^2^, up to a maximum of 6.6 times (Table 1).

At individual sampling points, the activity concentrations of ^137^Cs in the surface 30 cm layer of arable chernozem varied from ≈330 to 750 Bq kg^−1^ despite their insignificant distance from each other, which indicated a microfocal distribution of the radionuclide in soil. Such microfocal spatial heterogeneity of soil contamination with ^137^Cs, initially formed during the atmospheric Chernobyl precipitation onto terrestrial ecosystems, persists on agricultural lands of the PRH area even 35–40 years after the accident, with a coefficient of variation (C_v_) of 20%. Similar patterns of spatial variability in ^137^Cs accumulation levels in arable soils of the PRP area were noted in earlier observations by Golosov et al. in 1999 [32], in the recent study of Zhidkin et al. [25], and have also been recorded in remote croplands of Central Europe following the Chernobyl fallout [33,34]. Apparently, the long-term preservation of microspatial heterogeneity in the distribution of ^137^Cs on agricultural lands can be recognized as a characteristic feature of areas with accidental radioactive fallout.

**Figure 3:**
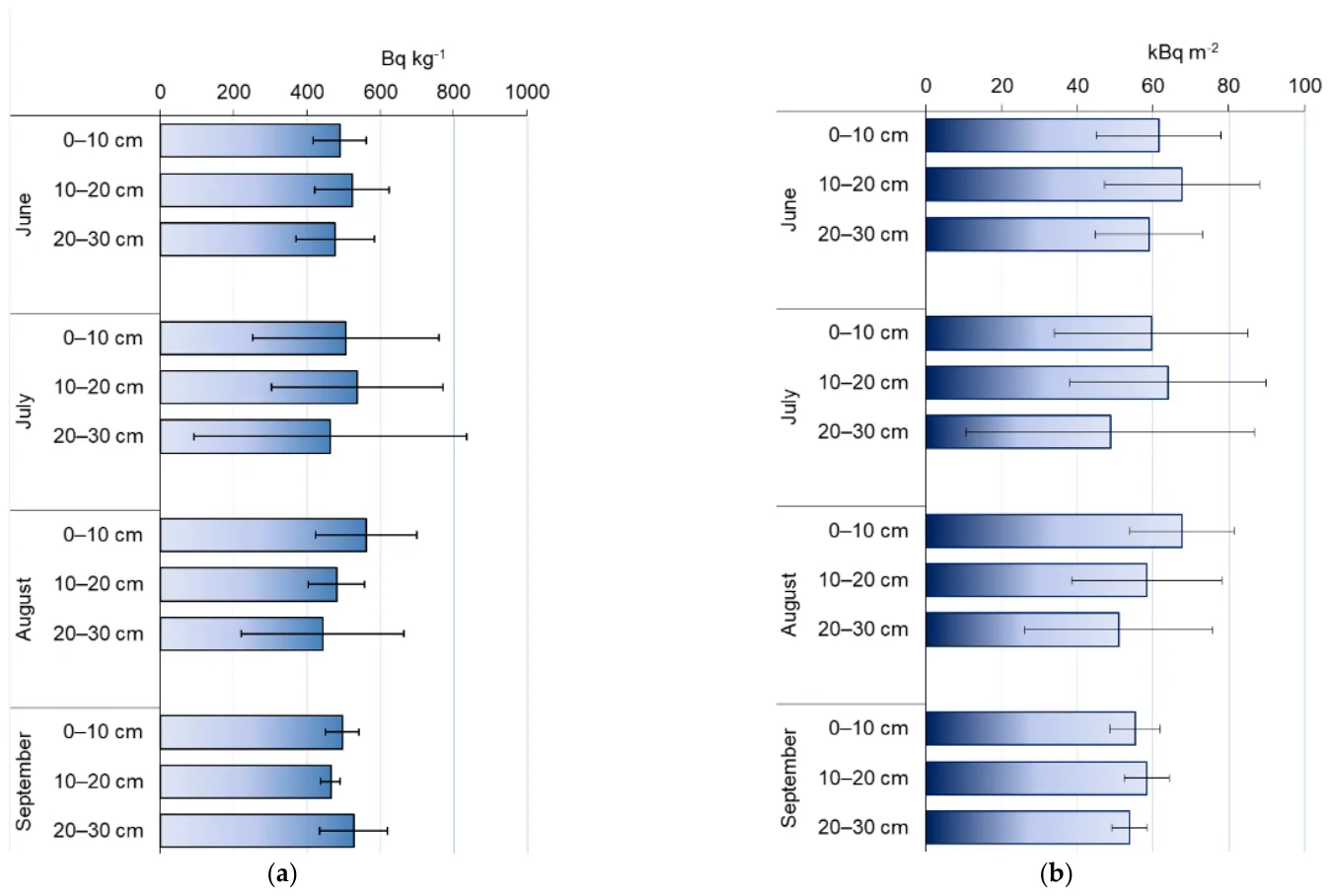
Vertical distribution of ^137^Cs in the 0–30 cm topsoil layer of arable chernozem of the PRH at different observation periods: (**a**) activity concentrations, Bq kg^−1^, (**b**) inventories, kBq m^−2^ (number of repetitions = 3).

The vertical distribution of ^137^Cs throughout the Ap^/^ (0–8 cm)–Ap^//^ (8–21 cm)–Aop (21–30 cm) horizons was more uniform than the spatial distribution: the maximum difference in activity concentrations between the mean values for the layers 0–10, 10–20, and 20–30 cm did not exceed 120 Bq kg^−1^, i.e., less than 25% (in August) (Figure 3). The greatest uncertainty in the levels of ^137^Cs content was observed in the layer 20–30 cm—both due to incomplete homogenization of the material during deep remedial plowing in 1986 and due to the possible inclusion in this layer of the underlying A horizon, which has an insignificant radionuclide content. In general, the distribution of ^137^Cs activity concentrations and inventories in the stratified core of the plow and old plowed horizons was rather uniform, without any seasonal dynamics of radionuclide redistribution.

In turn, ^137^Cs inventories were also distributed fairly evenly in the 0–10, 10–20, and 20–30 cm layers: 35, 35, and 30%, respectively. Homogenization of ^137^Cs content in plow layers is expected and often assumed a priori by many researchers, although there are few studies that empirically confirm this assumption. For example, in experiments with simulated deposition of ^137^Cs on soil and its subsequent manual plowing, it was shown that the radionuclide content within the surface 20 cm layer leveled off after three treatment cycles [35]. Also, a uniform distribution of ^137^Cs within the Ap horizon treated by a moldboard tillage technique was found seven years after the Fukushima Daiichi accident [36]. The obtained data confirm that after deep rehabilitation plowing and subsequent several decades of shallower agroturbation, the vertical distribution of ^137^Cs within stratified plow horizons (Ap^/^–Ap^//^–Aop) can be considered uniform, which is important to take into account when developing soil sampling and monitoring schemes in radioactively contaminated agricultural landscapes.

The absence of seasonal changes in the distribution pattern of ^137^Cs in the plow horizon is largely explained and indirectly confirms the concept of an extremely strong fixation of radiocaesium in clay chernozems, which determines the negligibly small desorption of the radionuclide from the soil exchange complex during atmospheric precipitation. The actual absence of an increase in ^137^Cs mobility during rainfall, including intensive or prolonged rainfall, was noted in a number of field observations in different ecological conditions [3,37,38,39]. Such data indicated the low importance of downward water migration of ^137^Cs in chernozem-like clayey soils during periods of seasonal precipitation.

A direct indicator of the low mobility of ^137^Cs in the studied soils was the radionuclide’s speciation in the main soil root zone (horizons Ap′ + Ap″ + Aop), which demonstrated the absolute predominance of the non-exchangeable (residual) fraction, firmly fixed in the soil matrix. The percentage ratio of water-soluble: exchangeable: mobile: acid-soluble: residual fractions was <0.1: 0.4: 0.9: 1.3: 97.4, which confirmed not only the stability of the total radionuclide pool, but also the extremely low proportion of mobile ^137^Cs that is potentially biologically available to agricultural crops in the clayey chernozems under study.

### 3.2. Root Uptake of ^137^Cs by Maize at Different Growth Stages and Intra-Planta Distribution of ^137^Cs

Unlike in soil, ^137^Cs activity concentrations in maize biomass varied significantly throughout crop ontogenesis from leaf development to ripening (Table 2). The ^137^Cs content in total biomass increased between growth stages S1 and S2 during leaf development and stem elongation, when the crop was at the peak of physiological activity, but then gradually declined by approximately twofold at the S3 and S4 stages during the periods of appearance and development of generative organs. However, it should be noted that in the maize agrosystem in the PRH area, seasonal fluctuations in ^137^Cs content in total biomass were only evident at the trend level without confirmation of their statistical significance. Similar results with uneven accumulation of ^137^Cs by plants during the vegetation period, but without certain patterns, were obtained in field observations for the above- and belowground parts of berry plants and medicinal herbs, as well as for higher vascular macrophytes in areas of the Ukrainian Polesye affected by Chernobyl fallout [18,40] or for leaves and branches of konara oak (*Quercus serrata*) in the Fukushima region [41].

The trends in seasonal changes in ^137^Cs activity concentrations in the organs of maize were more clearly expressed when considering the shoots and roots separately. Changes in ^137^Cs activity concentrations during growth were most clearly expressed and statistically confirmed. The maximum ^137^Cs activity concentrations were recorded in July at stage S2 (stem elongation) and the minimum values—in September at stage S4 (ripening). The difference between average values was as high as 2.5-fold. This would seem to confirm the concept of the “dilution effect” of ^137^Cs activity concentrations as plant biomass increases, which has been proposed by several authors [18,19,27,41,42]. But in fact, the process seemed to be more complicated: a significant decrease in ^137^Cs activity concentration occurred at the beginning of the cob development phase (S3), which was caused, most likely, not by a simple “dilution” of the radionuclide in the growing biomass, but by a change in biomass structure when the cobs appeared.

In vegetative organs, an increase in ^137^Cs activity concentrations was generally observed during the period of intensive growth of stems and leaves in June and July (S1–S2); the lowest values were observed in August upon entering the generative phase (S3). A similar pattern of seasonal rise and fall in ^137^Cs activity concentrations in green biomass seems to be characteristic of annual plants with an intermittent vegetation cycle, including agricultural crops [3,42,43,44], regardless of the current year’s weather conditions (air temperature or rainfall) [39]. However, in September, at maturity and full ripeness (S4), the radionuclide content in the stems and leaves of maize increased slightly again (at a statistically insignificant level). Thus, no signs of a decrease in the ^137^Cs content in stems and leaves as they aged were detected, as observed by some authors using woody plant species [19,45,46,47].

For maize’s generative organs—cobs and tassels—no statistically supported fluctuations in the ^137^Cs accumulation level were found from stage S3 (the beginning of the kernel’s formation) to stage S4 (full crop maturity).

Variability in the average ^137^Cs activity concentrations in the belowground biomass of maize was also weakly expressed. Only in the last observation period (S4) did activity concentrations of ^137^Cs decrease with statistical significance, presumably due to a reduction in metabolic activity and gradual dieback of the middle and especially fine roots.

In general, when comparing seasonal fluctuations of ^137^Cs activity concentrations in the above- and belowground parts of maize, relatively independent seasonal trends could be observed. The overall picture of seasonal changes in the ^137^Cs content in maize was apparently associated with changes in the structure of the biomass at different stages of growth, of which seasonal fluctuations in the levels of radiocaesium accumulation in individual plant organs were superimposed.

Finally, before the harvest in September, ^137^Cs activity concentrations in aerial parts of maize decreased in the following order: leaves > tassels, cob husks > cob stalks, stems > kernels, with the maximum difference in values being approximately tenfold. The phenomenon of effective protection of the grain of plants of the Gramineae family against the accumulation of radioactive elements is well known and has been confirmed in many previous studies [3,4,22,40,48,49,50]. There is also a more general hypothesis that vegetative organs protect maize kernels from negative abiotic factors, in particular, heat stress [51]. However, the differentiation of ^137^Cs accumulation inside the cob revealed in the present study (at the stage of ripeness S4, the ratios of activity concentrations in cob were: kernel: stalk: husks = 1: 3: 4), as well as the four times higher radiocaesium content in maize’s male generative organs (tassels) compared with that in the female ones (cobs), represents a rather new and interesting fact.

In the belowground part of maize by the end of the growing season, the following distribution of ^137^Cs activity concentrations was observed: fine roots > medium roots > brace roots, root necks. The difference in the accumulation of the radionuclide between the fine roots and the root necks in August (S3) and September (S4) was 7–13 times. The highest ^137^Cs activity concentrations were associated with fine roots, which have the greatest absorption capacity and supply plants with nutrients from the soil. It can be assumed that at S1 and S2 stages of phenological development, fine roots of maize were also characterized by significantly higher rates of ^137^Cs accumulation than medium ones, but the difficulty of collecting representative samples during the juvenile period did not allow differentiation of the belowground biomass by root size for these stages. At growth stages S3 and S4 after the emergence of brace roots, they were characterized by parameters of ^137^Cs accumulation relatively similar to those for the root necks and stems of maize.

The ability of the roots of some plant species to act as a biological barrier for ^137^Cs has been confirmed in several field observations and model experiments [3,4,49,52,53], and it is reasonable to assume that the rhizofiltration effect, that is, the ability to relatively concentrate the ecotoxicant coming from the soil inside or on the surface of cells, is achieved due to the fine roots. They are the first to interact with the soil in the process of root uptake of essential and non-essential nutrients. At the physiological level, the empirically observed accumulation of ^137^Cs in fine maize roots may presumably be associated with the manifestation of an “avoidance strategy,” which is used by plants to protect stress-sensitive photosynthetic tissues from the accumulation of pollutants [54].

An important, applied aspect of the obtained data on the concentrations of ^137^Cs activity in the grain of the Pioneer maize variety (hybrid P7515), grown in the fields of the PRH, was the conclusion that this product fully complied with the sanitary-hygienic requirements of the Russian Federation, which amount to 60 Bq kg^−1^ dry weight [55]. In turn, the regulations on the permissible ^137^Cs accumulation in stems and leaves of maize are not currently in force. The requirements of the national veterinary regulations for livestock feed were in effect until 2016 [56], which allowed radionuclides to accumulate up to ≤80 kg dry weight^−1^, and can be used as an estimate. In comparison, the stems and leaves of the studied maize, which were characterized by the highest values of ^137^Cs activity concentration among the aboveground parts of the biomass, still, like the grain, fully satisfied the regulatory requirements.

### 3.3. Comparative Analysis of ^137^Cs Activity Concentrations and Basic Growth and Development Parameters of Maize at Different Growth Stages

During the observation period, the maize plants underwent significant changes in growth and phenological development indicators. The height of the aerial part of the crop increased sixfold and its biomass more than 300-fold; in the belowground biomass, the same parameters changed by factors of 3 and 14, respectively (Table 3). The greatest rate of maize shoot’s linear increment was observed in June–July during the period of leaf unfolding between growth stages S1 and S2 (≈5 cm per day on average), while the increase in aboveground biomass was most pronounced in August–September during fruiting and ripening between growth stages S3 and S4 (≈66 g m^−2^ per day on average). Maize roots were mostly located in the Ah′ + Ap″ horizons, forming a rhizosphere space about 20 cm deep by July, while the belowground biomass increment continued from July to August (≈7 g m^−2^ per day on average), changing to a decrease in biomass by September when some of the roots died off. Nevertheless, the total biomass of maize increased steadily over the whole observation period, with its dynamics strongly influenced by the growth dynamics of the aerial parts of the crop (correlation coefficient r^2^ = 0.99).

In addition to changes in biometric traits of maize, the biomass structure changed significantly during the growing season, especially when generative organs appeared and developed. The ratio of above- to belowground parts of maize biomass also changed: if in June at growth stage S1 the roots dominated the shoots at a ratio of 3:2, then from July until the end of the growing season at stages S2–S4 the belowground biomass was in a ratio of 1:5–1:6 to the aboveground biomass. These structural changes largely determined the seasonal variability in dry matter content. Thus, increased growth of vegetative mass of maize in July was accompanied by a 1.5–2-fold decrease in dry matter content both in the shoots and in the roots, but after cob formation, this indicator significantly increased until September (twice the initial estimate for total biomass).

The seasonal variability of ash content was different and revealed a gradual decrease in the percentage of mineral nutrients in the above- and belowground organs (approximately threefold and twofold, respectively), which corresponded to a downward trend in transpiration rates from seedling to ripeness [57]. Zhang et al. [58] considered that the prevalence of root biomass at an early stage of plant ontogenesis allows maize roots to absorb nutrients better, which contributes to the successful formation of shoot organs, while the reduced intensity of root growth in the later stages of crop development contributes to the formation of dry matter content in the shoots. The content of potassium in maize (according to ^40^K) changed in close correlation with ash content (r^2^ = 0.75), reflecting its essential contribution to the total flux of nutrients from soils to plants.

When seasonal fluctuations of the levels of ^137^Cs accumulation in maize biomass were compared with variations in the productivity of shoots and roots, the dry matter content, or ^40^K activity concentration, no co-directional or opposite pairwise correlations were found. There was only a moderate positive correlation between ^137^Cs activity concentrations and ash content in the total biomass of maize (r^2^ = 0.57), most likely testifying to the passive involvement of the radionuclide in metabolism during the periods of intensive root uptake of nutrients.

**Figure 4:**
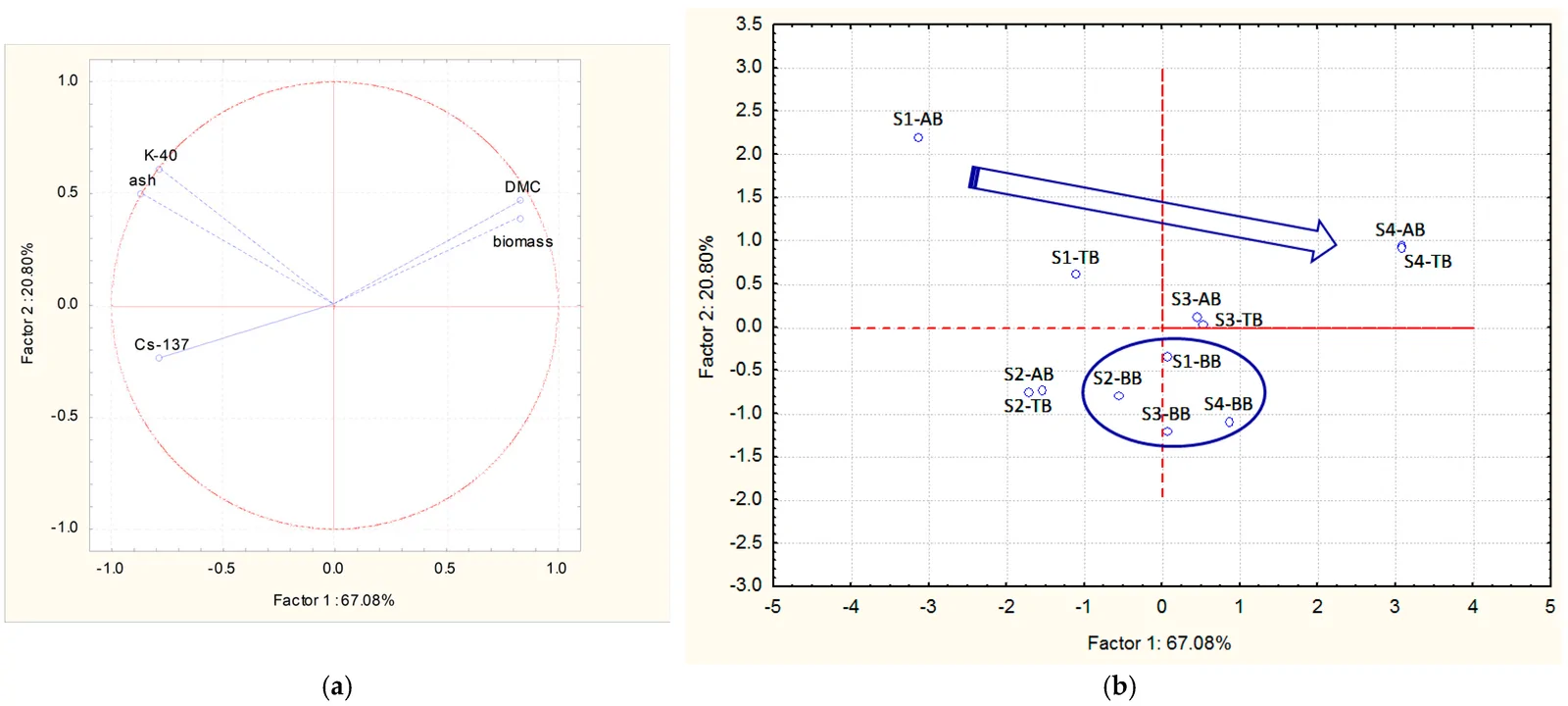
Projection of biomass (g m^−2^), dry matter content (DMC) (%), ash content (%), ^40^K and ^137^Cs activity concentration (Bq kg^−1^) values in aboveground biomass (AB), belowground biomass (BB) and total biomass (TB) of maize cultivated in the PRH area at crop growth stages S1–S4, obtained from the PCA. Biplot of factors (**a**) and cases (**b**). The percentage of total variance is indicated near principal component axes.

In turn, multi-factor analysis with the ordination of features along axes one and two of the principal components confirmed the specificity of ^137^Cs accumulation in maize, which was reflected in the opposition between its content and biomass productivity, as well as the overall consumption of nutrients during the growing season (Figure 4a), explaining 88% of the variance. The characteristics of total and aboveground biomass of maize shifted along the first ordination axis, closely related to biomass growth and dry matter formation as the crop passed through ontogenetic stages, while belowground biomass characteristics remained relatively stable and separated from the varying characteristics of aboveground biomass along the second ordination axis (Figure 4b). Similar changes in the structure of maize biomass at the stages of ontogenesis were in good agreement with the above-described seasonal dynamics of ^137^Cs activity concentrations in the total, above- and belowground biomass.

According to PCA imaging data, the most notable result was the distinction between ^137^Cs and ^40^K, as well as ash content, along the second ordination axis. While the seasonal trend of ^40^K content in maize, as expected, completely coincided with the dynamics of the general accumulation of mineral elements, ^137^Cs showed specific accumulation in biomass, mainly due to suppression of its translocation from roots to shoots. In the modern literature, the question of the similarity or difference between ^137^Cs and potassium in the processes of plant nutrition is controversial. Due to the similarity of the chemical properties of potassium and ^137^Cs, as well as the fact that the main mechanism for the penetration of Cs+ into plant cells is the non-specific cation channels common to monovalent ions [3,43,59,60], a number of researchers have drawn analogies between these elements [19,61,62,63]. Others point out that the biogeochemical cycles of ^137^Cs and potassium in the “soil–plant” system are not analogous [43,60,64,65,66,67]. There is also experimental evidence that at a high available potassium content in soil, e.g., ≥1 mmol l^−1^ or kg^−1^ (in the studied arable chernozems of the PRH area K_exch_ is 10–18 mmol kg^−1^ [26]), root uptake of ^137^Cs actually does not correlate with potassium [68,69,70]. It is currently believed that ^137^Cs may penetrate into the plant roots by high- and low-affinity types of K^+^ transporters, but the elements are in complex nonlinear relationships that depend on both their concentration in the soil solution and the presence of other mono- and divalent ions [68]. The data obtained in this study can be considered intermediate between the two positions: passive involvement of ^137^Cs in maize biomass during periods of intensive consumption of nutrients was detected, but its distribution in the plant organs did not coincide with the distribution of potassium.

**Figure 5:**
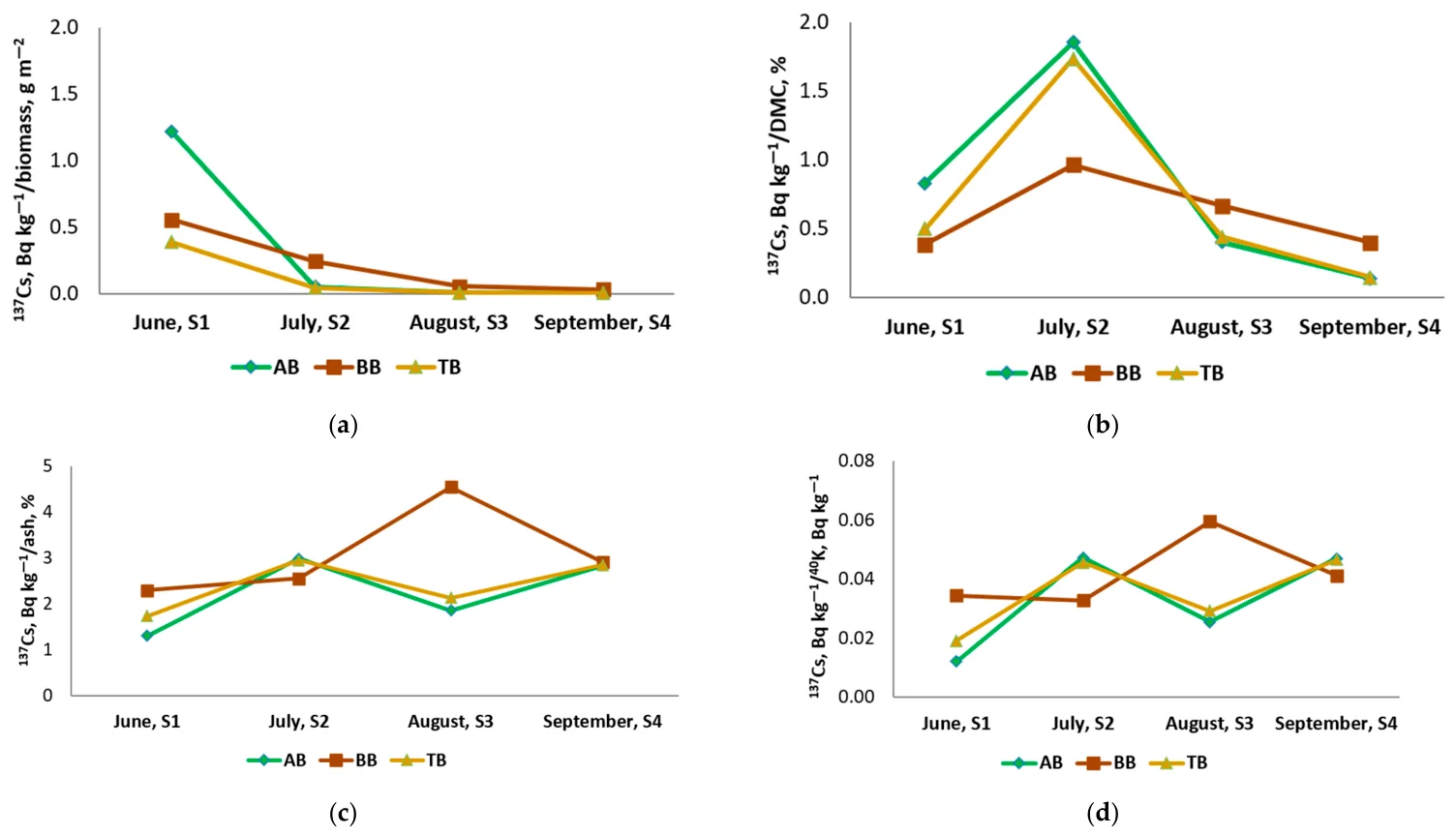
The ratio of the of ^137^Cs activity concentrations (Bq kg^−1^) in the total (TB), above- (AB) and belowground (BB) biomass to the characteristics of: (**a**) biomass (g m^−2^); (**b**) dry matter content (DMC, %); (**c**) ash content (%); (**d**) activity concentrations of ^40^K (Bq kg^−1^) at different stages of maize growth in the PRH area.

A paired analysis of ^137^Cs activity concentrations in the crop biomass and basic growth and phenological development parameters of maize did reveal certain complicated patterns (Figure 5). The conditional share of ^137^Cs relative to total and aboveground biomass was indeed highest in June (S1) and lowest in September (S4), but the ^137^Cs-to-dry matter content ratio was highest in July (S2) and lowest in September (S4). The ratio of ^137^Cs-to-ash or ^40^K ratio was highest in July (S3) and September (S4) and lowest in June (S1).

In the belowground part of maize, similar trends were observed for ^137^Cs-to-biomass and ^137^Cs-to-dry matter content ratios, but relatively smoothed seasonal fluctuations were observed in the ^137^Cs-to-ash and ^137^Cs-to-^40^K ratios, reaching a maximum in August (S3). Thus, the trigger for the variability of ^137^Cs transfer parameters from soil to maize plants was probably the ratio of intensities of the main physiological processes—photosynthesis, consumption of nutrients, biosynthesis, transport and decay of substances, emergence of generative organs, etc.—which changed as the crop progressed through the stages of ontogenesis.

### 3.4. TF and TLC Values at Different Growth Stages of Maize

A valuable indicator of ^137^Cs biological migration is TF, which reflects the intensity of element consumption by roots and can be used as a predictor in a phylogenetic model of radionuclide transfer from soil to plants [14]. The TF values obtained in this study varied during the growing season, reflecting fluctuations in ^137^Cs content in maize and its separate organs (Table 4). While the fluctuations of TF values in aboveground biomass and its fractions showed no statistically significant differences among growth stages, the seasonal course of TF parameters in the belowground part and especially in roots showed a marked increase from the leaf development stage in June (S1) to the cob development stage in August (S3), followed by a reduction to baseline TF_roots_ values at maturation (S4). Seasonal variations in TF values in the total biomass of maize were “dampened” by the predominance of the aerial part in its structure.

The seasonal variations in TF of ^137^Cs for the main consumed products of maize cultivation—kernels (grain) and green matter (stems + leaves + young cobs) for fodder—were also not statistically confirmed. The quantitative TF values obtained for grain as well as stems + leaves of maize grown in the semi-arid PRH area were in reasonable agreement and fell within the range of variation in the average TF values recommended by the IAEA for temperate [11] and arid [12] areas, estimated as 1.2 × 10^−2^ and 8.0 × 10^−4^ (grain), and 2.2 × 10^−2^ and 3.8 × 10^−3^ (stems and leaves), respectively. Thus, it can be assumed that for practical purposes of agricultural land-use planning in areas contaminated by condensed radioactive fallout, generalized or empirically determined regional TF values are an acceptable tool for decision-making.

The intensity of *intra-plant* transfer of radionuclides from belowground to aboveground organs is also an important feature of biological ^137^Cs migration, which is characterized by the TLC index. In particular, Zhu et al. [71] and Staunton et al. [72] suggested that differences in the behavior of radionuclides in “soil–plant” systems with different plant species may be determined not by the total root uptake of ^137^Cs, but by the specificity of their transfer from roots to shoots. Rather unexpectedly and notably, the TLC values were slightly greater than one at the early stages of maize growth (S1 and S2) and less than one at the reproductive stages (S3 and S4). So the predominant accumulation of ^137^Cs in the roots of plants from the Gramineae family, identified in a number of studies [4,48,72,73], is probably achieved during the ripening period, including the harvest stage.

### 3.5. Inventories of ^137^Cs in Maize, Balance of the Radionuclide in “Arable Chernozem–Maize” System, PRA Value

**Figure 6:**
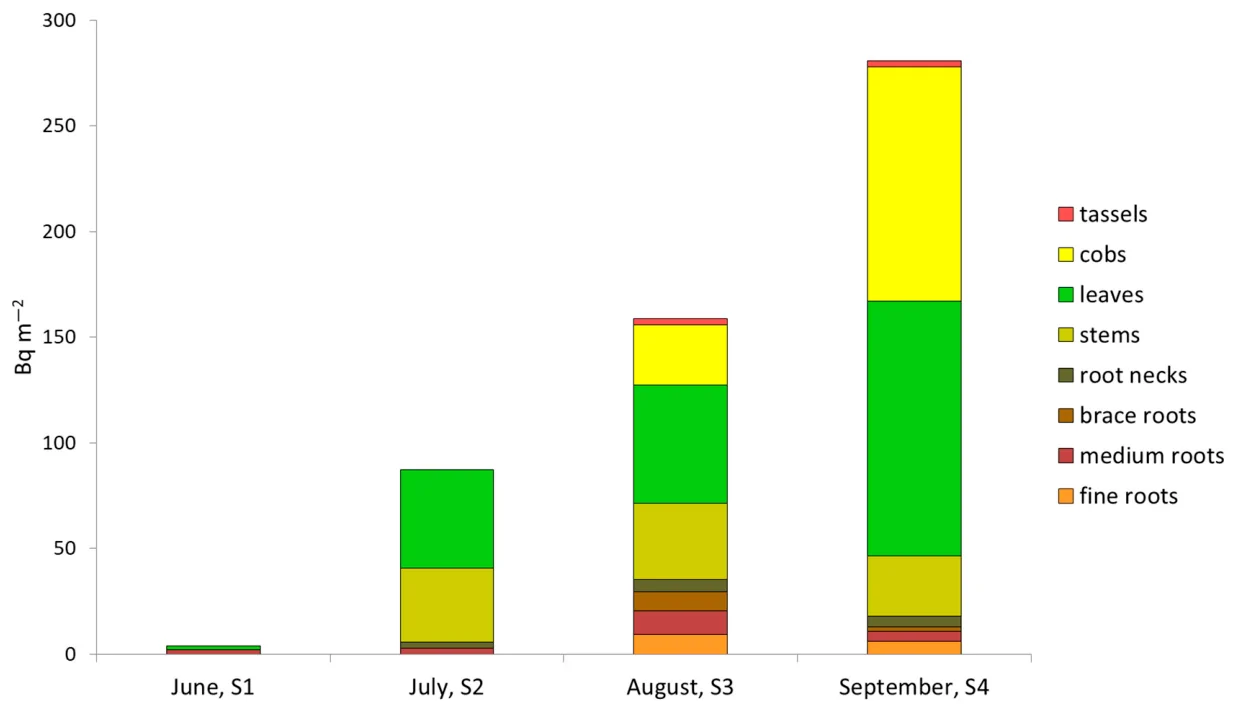
Inventories of ^137^Cs in maize cultivated in the PRH area at different growth stages, Bq m^−2^.

Owing to a steady increase in maize biomass, total ^137^Cs inventories involved in the crop also increased continuously from the initial observation date in June until the plants reached full maturity in September, regardless of seasonal variations in the radionuclide content in the above- or belowground parts (Figure 6). From the developmental stage of three–five leaves (S1) to ripening (S4), total ^137^Cs inventories in maize biomass increased from 4 to 280 Bq m^−2^, i.e., ≈80-fold, while the biomass itself grew by 115-fold. Consequently, in the process of plant growth, ^137^Cs was continuously consumed by the crop even at the late stage of ripeness, and not only redistributed between tissues, as was noted in several studies based on comparative measurements of the radionuclide activity concentrations in young and old leaves [18,42,73].

At all stages of maize growth, the main pool of ^137^Cs was associated with leaves, where 35–54% of the total radionuclide inventories were stored. The subdominant contribution to ^137^Cs inventories at the end of the vegetative phase of plant development was made by stems (40%), and at the end of the reproductive phase, by maize cobs (40%). Tassels, which accumulated approximately 1% of the total ^137^Cs inventories, served as the least significant depot for the accumulation of the radionuclide, corresponding to their share in the total maize biomass.

From the point of view of the possible organization of phytoremediation measures, it is important that in the period from July to September, at growth stages S2–S4, the ^137^Cs inventories in aerial parts exceeded those in the roots by 4–15 times. Thus, most of the radionuclide contained in maize aboveground biomass can potentially be removed with the harvest. However, in the overall balance of ^137^Cs inventories in the system “arable chernozem–maize,” the radionuclide removal with the annual harvest was not more than 0.2%, while more than 99.8% remained deposited in the soil (Table 5). Such a distribution of ^137^Cs was apparently determined by both the physiological discrimination of its root uptake by maize and the very low bioavailability of the radionuclide from neutral clayey soils [15].

In fact, under the conditions of a particular “soil–plant” system, the measure of the true bioavailability of radionuclides, heavy metals, and rare earth metals can be precisely the amount of the element transferred from the soil to the biomass [74,75]. For the studied system “clayey agrochernozems–maize” of the PRH area, the bioaccumulation of ^137^Cs, incorporated by the end of the growing season into the total biomass, was significantly less than the inventories of its potentially bioavailable forms. It was estimated to be less than the sum of water-soluble and exchangeable forms (0.7 kBq m^−2^) by approximately 1.5 times; water-soluble, exchangeable and mobile forms (2.2 kBq m^−2^) by approximately 8 times; water-soluble, exchangeable, mobile, and partially accessible to plants acid-soluble forms (4.3 kBq m^−2^) by approximately 15 times. Therefore, the results of the current study showed that when analyzing the reasons for low ^137^Cs TF values, one should not underestimate the importance of the biological factor of suppressing root uptake of the radionuclide.

In general, the PRA value of 0.2% in an agrosystem of maize cultivated on radioactively contaminated clayey chernozem seemed too low even in comparison with the natural ^137^Cs decay rate, which is 2.3% per year (according to the radiocaesium decay constant). On the other hand, the low bioavailability of ^137^Cs for maize determined the hygienic safety of products obtained in the PRH area and directly consumed by humans or transferred through cattle to human food chains. This makes it possible to confirm that maize belongs to the so-called “safe crops” at all stages of its growth, including both baby corn and fully ripe kernels.

## 4. Conclusions

The Chernobyl accident in 1986 became a trigger for large-scale studies of the behavior of ^137^Cs in the environment. Yet, the patterns of seasonal fluctuations in the radionuclide transfer from contaminated soils to agricultural crops still remain insufficiently studied. Since they are related to changes in the most important physiological and metabolic processes during plant phenological stages, they can clarify the specificity of biological migration of ^137^Cs.

For grain maize Pioneer (hybrid P7515) cultivated in the post-Chernobyl PRH area with a current ^137^Cs content of 176 ± 19 kBq m^−2^ (498 ± 57 Bq kg^−1^) in arable clayey Luvic Chernozem, the study showed low radionuclide root uptake throughout the growing season. From the development stage of three to five leaves to the stage of ripening, TF demonstrated statistically insignificant fluctuations (1.5 × 10^−2^–3.6 × 10^−2^) with a geometric mean of 2.2 × 10^−2^. In addition, asynchronous changes in ^137^Cs activity concentrations, and consequently, TF_AB_ and TF_BB_ values, were detected in the above- and belowground parts of maize during the growth and development of the crop. In the aerial parts as a whole, maximum ^137^Cs activity concentrations were observed at the end of the vegetative stage in July, decreasing to a minimum in August–September, when maize transitioned to the reproductive stage. All seasonal dynamics of ^137^Cs content in aboveground biomass were associated with leaves and stems, while the generative organs (cobs and tassels) showed virtually constant levels of radionuclide accumulation from the beginning of kernel development until physiological maturity. At the same time, in the belowground part of maize, an increase in ^137^Cs levels was noted in August, which coincided with the period of the highest relative representation of the fraction of fine roots. The relative concentration of ^137^Cs in fine roots compared with that in larger ones suggested the presence of a rhizofiltration effect, which is one of the mechanisms by which plants protect themselves from ecotoxicants.

Significant differentiation of ^137^Cs accumulation levels in individual organs of maize revealed in the present study, which were established at the juvenile growth stage and maintained during the growing season, as well as the specificity of seasonal variability of radiocaesium in plants in comparison with total biomass, dry matter, nutrients, and ^40^K might be another reason to pay more attention to the study of ^137^Cs root uptake and translocation during ontogenesis. Such investigations seem to be useful for phylogenetic predictions of ^137^Cs transfer to crops, which are necessary for land-use decisions on radioactively contaminated agricultural lands.

Although the current study was based on the results of one growth season, with one maize hybrid and one soil type, it can be assumed that the obtained patterns of ^137^Cs root uptake during crop development were reasonably representative of the “clay neutral soils–maize” system, since the weather conditions during the observations were close to the average long-term characteristics, the chernozem could be considered a typical representative of a group of similar soils, and varietal differences between crops are less important than species differences. Overall, it can be assumed that the key to establishing the parameters of biogenic migration of ^137^Cs in the “soil–plant” system lies in the systematic recording of the seasonal dynamics of its accumulation in individual plant organs simultaneously with monitoring the structure of the biomass, which changes significantly precisely at the seasonal stages of ontogenesis.

As regards the assessment of the current possibility of commercial maize cultivation on croplands of the PRH area, the low bioavailability of the radionuclide from clayey chernozem, as well as pronounced discrimination of its penetration into kernels (using the example of the studied grain maize Pioneer, hybrid P7515) allows the proposed approach of actively using ^137^Cs-contaminated sites in agriculture based on the cultivation of “safe crops” to be recognized as environmentally sound and acceptable.

## Figures and Tables

**Table 1:** Content of ^137^Cs in 30 cm topsoil layer of arable chernozem of the maize agrosystem in the PRH area (number of repetitions = 3).

Month, Stage of Maize Growth	Mean ± CI ^1^	Median	Range	C_v_ ^2^, %
Activity concentration, Bq kg^−1^				
June, S1	497 ± 75 ^a 3^	472	448–573	13
July, S2	504 ± 244 ^a^	433	333–747	43
August, S3	497 ± 65 ^a^	465	463–563	12
September, S4	493 ± 25 ^a^	491	472–516	4
June–September, S1–S4	498 ± 57 ^a^	472	333–747	20
Inventory, kBq m^−2^				
June, S1	188 ± 45 ^a^	181	152–231	21
July, S2	172 ± 75 ^a^	151	120–246	38
August, S3	177 ± 9 ^a^	174	171–186	4
September, S4	167 ± 5 ^a^	169	162–169	2
June–September, S1–S4	176 ± 19 ^a^	171	120–246	19

^1^ Mean ± CI is arithmetic mean ± confidence interval at α < 0.05; ^2^ C_v_ is coefficient of variation; ^3^ the same letter ‘a’ next to the data in the column indicates statistically insignificant differences between the mean values after Tukey’s honestly significant difference multiple comparison at α < 0.05.

**Table 2:** ^137^Cs activity concentrations in maize cultivated in the PRH area at different growth stages, Bq kg^−1^ (number of repetitions = 3).

Plant Part	June, S1	July, S2	August, S3	September, S4
TB ^1^	11.8 ± 1.7 ^a 4^	17.9 ± 2.2 ^a^	9.7 ± 2.8 ^a^	7.6 ± 2.4 ^a^
AB ^2^, including	13.8 ± 2.1 ^a^	18.7 ± 2.4 ^b^	9.0 ± 3.1 ^ac^	7.6 ± 2.5 ^c^
stems		16.4 ± 1.3 ^a^	5.0 ± 2.1 ^b^	7.4 ± 1.5 ^b^
leaves		31.6 ± 2.8 ^a^	21.3 ± 2.3 ^b^	31.0 ± 9.0 ^ab^
cobs			7.7 ± 5.5 ^a^	4.1 ± 1.6 ^a^
cob husks				12.6 ± 3.3
cob stalks				8.6 ± 3.0
kernels (grain)				3.1 ± 1.3
tassels			15.8 ± 2.9 ^a^	14.8 ± 3.8 ^a^
BB ^3^, including	10.7 ± 1.6 ^a^	11.7 ± 0.9 ^a^	13.6 ± 1.7 ^a^	7.3 ± 1.1 ^b^
root necks		10.3 ± 0.9 ^a^	5.0 ± 0.6 ^b^	3.8 ± 0.7 ^b^
roots, including		13.9 ± 1.5 ^a^	20.8 ± 2.5 ^b^	11.1 ± 1.6 ^a^
brace roots			12.4 ± 2.1 ^a^	4.6 ± 0.9 ^b^
medium roots			20.5 ± 1.2 ^a^	11.2 ± 1.2 ^b^
fine roots			65.8 ± 9.2 ^a^	22.4 ± 3.4 ^b^

^1^ TB—total biomass; ^2^ AB—aboveground biomass; ^3^ BB—belowground biomass; ^4^ the same letters next to the data in the rows indicate statistically insignificant differences, and different letters indicate significant differences between the mean values after Tukey’s honestly significant difference multiple comparison at α < 0.05.

**Table 3:** General characteristics of maize biomass, ash content and ^40^K activity concentrations in the crop at different growth stages in the PRH area (number of repetitions = 3).

Characteristics	June, S1	July, S2	August, S3	September, S4
Height of shoots, cm	38 ± 3 ^a^	163 ± 12 ^b^	252 ± 6 ^c^	240 ± 9 ^c^
Root length, cm	9 ± 1 ^a^	21 ± 2 ^b^	23 ± 3 ^b^	23 ± 1 ^b^
Dry biomass, g m^−2^				
TB ^1^	30.7 ± 2.7 ^a 4^	412.0 ± 199.3 ^b^	1622.8 ± 112.4 ^c^	3576.7 ± 217.0 ^d^
AB ^2^, including	11.3 ± 1.3 ^a^	364.0 ± 224.2 ^b^	1365.5 ± 128.5 ^c^	3339.3 ± 229.6 ^d^
stems		218.4 ± 134.5 ^a^	723.3 ± 173.3 ^b^	412.0 ± 74.5 ^c^
leaves		145.6 ± 89.7 ^a^	266.0 ± 12.0 ^b^	388.7 ± 26.4 ^c^
cobs			359.3 ± 130.4 ^a^	2521.3 ± 284.8 ^b^
cob husks				138.7 ± 35.2
cob stalks				288.0 ± 33.5
kernels (grain)				2233.3 ± 332.7
tassels			16.8 ± 6.9 ^a^	17.3 ± 6.9 ^a^
BB ^3^, including	19.3 ± 3.5 ^a^	48.0 ± 10.4 ^b^	257.3 ± 27.0 ^c^	237.3 ± 32.1 ^c^
root necks		29.5 ± 6.4 ^a^	116.1 ± 12.2 ^b^	121.0 ± 16.4 ^b^
roots, including		18.5 ± 4.0 ^a^	141.2 ± 6.3 ^b^	116.4 ± 5.5 ^c^
brace roots			74.4 ± 7.8 ^a^	48.14 ± 6.51 ^b^
medium roots			52.6 ± 5.5 ^a^	41.2 ± 5.6 ^b^
fine roots			14.2 ± 1.5 ^a^	27.0 ± 3.7 ^b^
Dry matter content, %				
TB	23.8 ± 7.2 ^ab^	10.3 ± 6.6 ^a^	22.2 ± 4.6 ^b^	52.9 ± 11.0 ^c^
AB, including	16.7 ± 6.5 ^ab^	10.1 ± 7.0 ^a^	22.5 ± 4.6 ^b^	55.2 ± 11.5 ^c^
stems		10.5 ± 3.7 ^a^	22.5 ± 8.9 ^a^	16.5 ± 3.0 ^a^
leaves		18.7 ± 4.4 ^a^	28.9 ± 3.8 ^b^	22.4 ± 1.7 ^a^
cobs			16.9 ± 6.5 ^a^	65.9 ± 14.1 ^b^
cob husks				59.8 ± 5.9
cob stalks				44.3 ± 7.3
kernels (grain)				69.1 ± 12.7
tassels			44.0 ± 6.9 ^a^	78.8 ± 8.0 ^b^
BB, including	28.0 ± 7.6 ^a^	12.1 ± 3.4 ^b^	20.5 ± 4.7 ^a^	18.5 ± 3.4 ^ab^
root necks		10.8 ± 1.9 ^a^	22.9 ± 5.1 ^b^	19.4 ± 3.5 ^b^
roots, including		14.3 ± 2.9 ^a^	18.6 ± 2.4 ^a^	17.6 ± 3.5 ^a^
brace roots			22.2 ± 3.4 ^a^	20.8 ± 2.2 ^a^
medium roots			15.6 ± 3.0 ^a^	16.1 ± 2.1 ^a^
fine roots			10.8 ± 2.8 ^a^	14.2 ± 3.6 ^a^
Ash content, %				
TB	6.8 ± 0.9 ^a^	6.1 ± 0.1 ^a^	4.6 ± 0.6 ^b^	2.7 ± 0.2 ^c^
AB, including	10.5 ± 0.7 ^a^	6.3 ± 0.1 ^b^	4.8 ± 0.7 ^c^	2.7 ± 0.2 ^d^
stems		7.8 ± 1.2 ^a^	3.7 ± 0.8 ^b^	2.7 ± 0.3 ^b^
leaves		7.4 ± 0.8 ^a^	9.8 ± 0.5 ^b^	11.2 ± 0.4 ^c^
cobs			3.4 ± 0.6 ^a^	1.4 ± 0.2 ^b^
cob husks				3.4 ± 0.1
cob stalks				1.3 ± 0.5
kernels (grain)				1.3 ± 0.1
tassels			5.5 ± 0.3 ^a^	5.9 ± 0.2 ^a^
BB, including	4.6 ± 0.7	4.6 ± 0.6 ^a^	3.0 ± 0.3 ^b^	2.5 ± 0.2 ^b^
root necks		4.2 ± 0.8 ^a^	1.5 ± 0.2 ^b^	2.0 ± 0.1 ^c^
roots, including		5.2 ± 0.3 ^a^	4.2 ± 0.7 ^a^	3.1 ± 0.1 ^b^
brace roots			3.6 ± 0.5 ^a^	2.5 ± 0.1 ^b^
medium roots			4.4 ± 0.1 ^a^	3.0 ± 0.5 ^b^
fine roots			6.8 ± 0.3 ^a^	4.2 ± 0.3 ^b^
^40^K, Bq kg^−1^				
TB	623 ± 17 ^a^	393 ± 61 ^b^	334 ± 38 ^b^	163 ± 29 ^c^
AB, including	1154 ± 32 ^a^	661 ± 28 ^b^	348 ± 38 ^c^	162 ± 30 ^d^
stems		679 ± 11 ^a^	281 ± 33 ^b^	275 ± 65 ^b^
leaves		633 ± 16 ^a^	529 ± 90 ^ab^	448 ± 97 ^b^
cobs			366 ± 10 ^a^	103 ± 15 ^b^
cob husks				448 ± 161
cob stalks				139 ± 27
kernels (grain)				77 ± 4
tassels			410 ± 11 ^a^	77 ± 19 ^b^
BB, including	311 ± 8 ^a^	358 ± 16 ^b^	230 ± 42 ^c^	179 ± 20 ^c^
root necks		304 ± 7 ^a^	91 ± 50 ^b^	158 ± 16 ^c^
roots, including		444 ± 58 ^a^	343 ± 34 ^b^	200 ± 24 ^c^
brace roots			386 ± 28 ^a^	242 ± 52 ^b^
medium roots			311 ± 51 ^a^	240 ± 7 ^b^
fine roots			237 ± 6 ^a^	64 ± 2 ^b^

^1^ TB—total biomass; ^2^ AB—aboveground biomass; ^3^ BB—belowground biomass; ^4^ the same letters next to the data in the rows indicate statistically insignificant differences, and different letters indicate significant differences between the mean values after Tukey’s honestly significant difference multiple comparison at α < 0.05.

**Table 4:** TF and TLC values for maize agrosystem at different growth stages of the crop cultivated in the PRH area.

Parameter	June, S1	July, S2	August, S3	September, S4	June–September, S1–S4 ^1^
TF_TB_ ^2^	2.4 × 10^−2 a 5^	3.6 × 10^−2 a^	2.0 × 10^−2 a^	1.5 × 10^−2 a^	2.2 × 10^−2^
TF_AB_	2.8 × 10^−2 a^	3.7 × 10^−2 a^	1.8 × 10^−2 a^	1.5 × 10^−2 a^	2.3 × 10^−2^
TF_stems and leaves_	2.8 × 10^−2 a^	3.7 × 10^−2 a^	4.5 × 10^−2 a^	1.9 × 10^−2 a^	3.8 × 10^−2^
TF_cobs_			1.6 × 10^−2 a^	8.4 × 10^−3 a^	1.1 × 10^−2^
TF_kernels(grain)_				6.2 × 10^−3^	6.2 × 10^−3^
TF_BB_ ^4^	2.2 × 10^−2 a^	2.3 × 10^−2 abc^	2.7 × 10^−2 b^	1.5 × 10^−2 c^	2.1 × 10^−2^
TB_roots_	2.2 × 10^−2 a^	2.8 × 10^−2 ab^	4.2 × 10^−2 b^	2.2 × 10^−2 a^	3.0 × 10^−2^
TLC	1.10 ^a^	1.29 ^a^	0.47 ^b^	0.68 ^b^	0.82

^1^ geometric mean; ^2^ TB—total biomass; ^3^ AB—aboveground biomass; ^4^ BB—belowground biomass; ^5^ the same letters next to the data in the rows indicate statistically insignificant differences, and different letters indicate significant differences between the mean values after Tukey’s honestly significant difference multiple comparison at α < 0.05.

**Table 5:** Balance of ^137^Cs inventories in the “arable chernozem–maize” system in the PRH area before harvesting (September, S4).

Component	^137^Cs, kBq m^−2^	^137^Cs, %
Soil (0–30 cm)	167	99.8
TB ^1^	2.8 × 10^−1^	0.17
AB ^2^	2.6 × 10^−1^	0.16
BB ^3^	1.8 × 10^−2^	0.01

^1^ TB—total biomass; ^2^ AB—aboveground biomass; ^3^ BB—belowground biomass.

## Data Availability

The original data presented in the study are included in the article; further inquiries can be directed to the corresponding author.
